# Mitochondrial Modification Techniques and Ethical Issues

**DOI:** 10.3390/jcm6030025

**Published:** 2017-02-24

**Authors:** Lucía Gómez-Tatay, José M. Hernández-Andreu, Justo Aznar

**Affiliations:** 1 Escuela de Doctorado Universidad Católica de Valencia San Vicente Mártir, Valencia 46001, Spain; lucia.gomez@ucv.es; 2 Facultad de Medicina y Odontología, Universidad Católica de Valencia San Vicente Mártir, Departamento de Ciencias Médicas Básicas, Grupo de Medicina Molecular y Mitocondrial, Valencia 46001, Spain; jmiguel.hernandez@ucv.es; 3 Institute of Life Sciences, Universidad Católica de Valencia San Vicente Mártir, Valencia 46001, Spain

**Keywords:** mitochondrial disease, mitochondrial replacement, gene editing, ethics, pronuclear transfer, maternal spindle transfer, polar body transfer, CRISPR, TALENs

## Abstract

Current strategies for preventing the transmission of mitochondrial disease to offspring include techniques known as mitochondrial replacement and mitochondrial gene editing. This technology has already been applied in humans on several occasions, and the first baby with donor mitochondria has already been born. However, these techniques raise several ethical concerns, among which is the fact that they entail genetic modification of the germline, as well as presenting safety problems in relation to a possible mismatch between the nuclear and mitochondrial DNA, maternal mitochondrial DNA carryover, and the “reversion” phenomenon. In this essay, we discuss these questions, highlighting the advantages of some techniques over others from an ethical point of view, and we conclude that none of these are ready to be safely applied in humans.

## 1. Introduction

Mitochondria are organelles present in the cytoplasm of most eukaryotic cells. Although their main function is the production of cellular energy, they also play an important role in other cell processes, such as calcium signalling, regulation of cell metabolism, embryonic development and programmed cell death [1]. In addition, they are implicated in the pathogenesis of numerous diseases, in particular neurodegenerative disorders [2].

These organelles contain their own DNA, known as mitochondrial DNA (mtDNA) [3], which is a circular double-helix DNA molecule, which in humans contains 37 genes: 13 of these code for a polypeptide involved in the respiratory chain, 22 for transfer RNAs (tRNA) and two for ribosomal RNAs (rRNA), all responsible for the translation of these 13 peptides [4]. The mitochondrial electron transport chain is composed of freely moving respiratory complexes and mobile electron carriers that coexist with larger structures called respiratory supercomplexes [5].

Mitochondrial biogenesis and function is dual, depending on both the nuclear and mitochondrial genome. Thus, the replication of mtDNA, its packaging in nucleoids (DNA-protein complexes), and its transcription and translation are processes that depend, to a large extent, on nuclear-encoded proteins [6]. Furthermore, the processes of mitochondrial fusion and fission, which enable intermitochondrial cooperation and compartmentalisation of organelles, respectively, are controlled by products that come completely from the expression of the nDNA [7]. With respect to mitochondrial function, 79 of the 92 subunits that comprise the oxidative phosphorylation (OXPHOS) system are encoded by the nDNA [6]. Thus, primary respiratory chain defects may be due to hereditary alterations in the mtDNA (deletions, rearrangements or point mutations) or nDNA genes that encode subunits for this system, as well as somatic mutations resulting from the action of free radicals, which either directly damage the mtDNA or prevent correct repair of the damage [8].

Mitochondrial alterations cause a decrease in cellular energy that can affect different organs, expressing various clinical phenotypes [6], and can cause significant morbidity and mortality [9,10]. The prevalence of diseases due to mutations in mtDNA is approximately 1 per 5000 individuals, although it may be much higher in certain regions due to genetic founder mutations and high consanguinity [2]. Furthermore, one in 200 healthy individuals is a carrier of a pathogenic mitochondrial mutation that can affect the offspring of female carriers [11].

The proportion of mutant mtDNA can vary between tissues and over time. In the case of the most common point mutations, the disease manifests at cellular level if a threshold of 80%–90% mutated mitochondria is exceeded [12,13]. The proportion necessary for the disease to manifest varies depending on the mutation, the tissue and even on the individual, since environmental factors, physical exercise or the nuclear genetic load itself can also have an effect [14].

mtDNA is inherited exclusively from the mother, however, the level of heteroplasmy varies between individuals descended from the same mutant mtDNA mutation carrier mother. This is due to the “bottleneck” effect that occurs in mitochondrial transmission. Only a fraction of the mother’s mitochondria pass to the offspring, which explains the variation in the level of heteroplasmy between different generations and between siblings. This genetic drift has been thought to be random, but recent studies point towards differences in the behaviour of the mtDNA bottleneck, depending on the specific mtDNA mutation [15]. Knowing the expected probability for the heteroplasmy values in the offspring is important for genetic counselling of the future parents [16,17,18].

## 2. Treatment of Mitochondrial Diseases

Although vitamin supplements, drugs and physical exercise have been used as treatment in isolated cases and small clinical trials, there is currently no evidence on the effectiveness of these interventions on mitochondrial disorders [19], so new treatment approaches are being developed [2]. However, the highest expectations have been placed on two types of novel techniques that seem to have great potential for application, so that both women affected by a disease due to an alteration in their mtDNA and asymptomatic carriers can have children free from the mutation.

The first group is based on the use of healthy donor mitochondria. These are known as mitochondrial replacement techniques: maternal spindle transfer (MST), pronuclear transfer (PNT), and the most recent, polar body transfer (PBT). Although the first two were authorised for clinical use in the United Kingdom in October 2015 [20], the Human Fertilisation and Embryology Authority (HFEA) announced in June 2016 that the safety and efficacy of these techniques had to be confirmed before any medical centre could request a license to offer mitochondrial donation. To that end, a group of scientists was convened to review the latest advances in this respect [21]. The review by the panel of experts was published in November 2016, and recommends that “in specific circumstances, MST and PNT are cautiously adopted in clinical practice where inheritance of the disease is likely to cause death or serious disease and where there are no acceptable alternatives” [22]. After that, on 15 December 2016, the HFEA approved the use of mitochondrial donation in certain specific cases. Clinics wishing to offer these techniques to patients can now apply to the HFEA for permission to do so and then two committees will assess the suitability of the clinic and each particular clinic case [23].

These techniques are aimed at eradicating the maternal mtDNA in the individual’s cells. Nevertheless, there is always some carryover, which can mean that mutant mtDNA levels increase during subsequent development—a phenomenon known as “genetic instability” [24], “genetic drift” [25] or “reversion” [26]—and the disease reappears in later generations. A recent study calculated that, for a clinical threshold of 60%, reducing the mutant mtDNA transferred to below 5% would eradicate the disease forever in that lineage, while if this figure is exceeded, the likelihood that the disease will reappear in subsequent generations is high, so it is important to limit mutant mtDNA transmission to levels below 3% [27]. These low levels have already been achieved with the MST technique in primates [28] and human oocytes [29], with PNT in preimplantation human embryos [30], and with PBT in mouse oocytes and embryos [31]. However, these are probabilistic calculations, so no claims can be made with complete certainty in this respect. Moreover, there is no data of this type on the “reversion” phenomenon.

These techniques may be relatively easy to carry out and feasible for many clinics which can perform intracytoplasmic sperm injection (ICSI), if they can use donor eggs, but realizing them with the precision that is required for optimal results is not so simple. Thus, in the last scientific review of the safety and efficacy of mitochondrial donation to the HFEA, the panel of experts state that “key recommendations are conditional on a number of considerations, including a requirement for appropriate levels of skill being demonstrated by named practitioners within a named clinic, and relevant key performance indicators being met” [22]. In addition, in the report of the the Institute of Medicine (IOM) of the National Academies of Sciences, Engineering, and Medicine to the Food and Drug Administration (FDA), there is a section devoted to the “Expertise of Investigators and Centers” where they point out that “Most MRT approaches contemplated at present would involve highly intricate micro-manipulations of human gametes and/or embryos. Use of the techniques would therefore require operator skill, which evolves over time, varies from one individual to another, and resists specification in a protocol” ([32], p. 138). Finally, in the announcement of the HFEA on 15 December, it states that “HFEA’s Licence Committee will first assess a clinic’s suitability, looking at existing staff expertise, skill and experience at the clinic, as well as its equipment and general environment” [23].

The second group includes two gene editing techniques: CRISPR-Cas 9 (clustered regularly interspaced short palindromic repeats) and TALENs (transcription activator-like effector nucleases). These techniques, unlike the previous, have not been specifically designed to act on the mitochondria, but they can also be used to correct the mtDNA. Gene editing has been applied in some studies to reduce the levels of mutant mtDNA in heteroplasmic cells [33,34,35,36]. However, in order for its action to prevent the transgenerational transmission of mitochondrial diseases, it needs to act on the germline.

### 2.1. Mitochondrial Replacement Techniques

#### 2.1.1. Pronuclear Transfer

**Figure 1:**
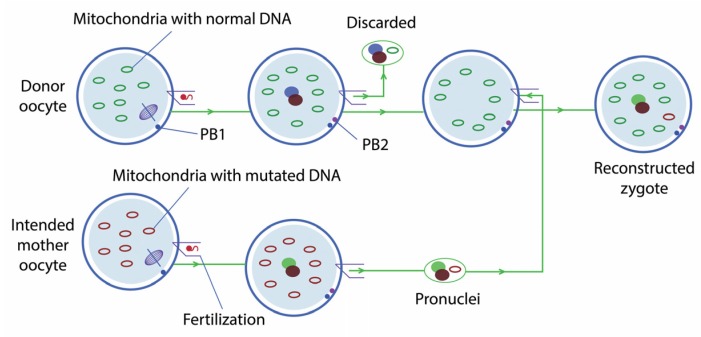
Pronuclear transfer.

PNT consists of performing in vitro fertilisation using the eggs of the affected woman—whose mitochondria contain mutant mtDNA—and the sperm of the future father, and subsequent extraction of the pronuclei on day 1 of development, leaving behind most of the mutated mitochondria. These pronuclei are transferred to an enucleated zygote with healthy mitochondria (Figure 1); they are transferred to an enucleated zygote, not an egg, since the developmental state must be the same. The hybrid zygote is then developed in vitro until it reaches an appropriate state for transfer to the uterus. Thus, this technique is not strictly preventive, since the gene transfer takes place once the zygote is produced.

Craven et al. applied this technique in embryos with an abnormal number of pronuclei, and succeeded in eliminating more than 98% of the maternal mitochondria [30], which, in principle, is sufficient to prevent clinical manifestation of the disease [37], and its transmission to subsequent generations [27]. PNT has also been applied in normally-fertilised human embryos, with a percentage of mtDNA carryover that did not exceed 5% in any case, and which in most embryos was less than 2% [25]. However, it was observed that, in a stem cell line derived from a blastocyst with 4% mtDNA carryover, the mother’s mtDNA gradually increased its proportion with respect to that of the donor. The causes of this reversion are unknown, although it is speculated (among other reasons) that one haplotype may have a replicative advantage over another in specific combinations [22]. The case of an infertile woman who had become pregnant with triplets using this technique was later published, although none of the foetuses reached full term [38]. 

#### 2.1.2. Maternal Spindle Transfer

**Figure 2:**
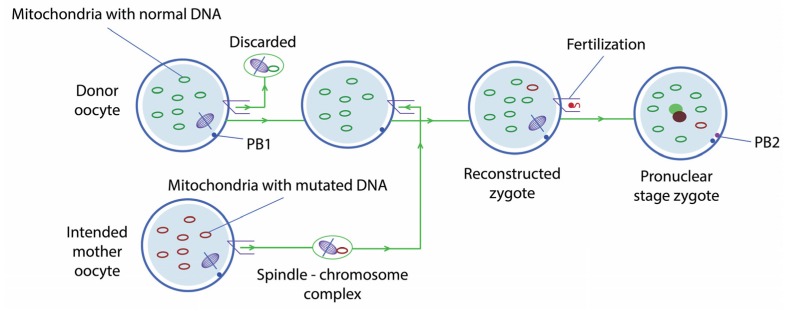
Maternal spindle transfer.

MST consists in extracting the chromosomes in metaphase II from the mother’s egg—whose mtDNA has some mutation—to then transfer them to a healthy donor egg, in which the chromosomes have been removed. The hybrid egg is fertilised in vitro and then transferred to the mother’s uterus (Figure 2). This technique, however, is strictly preventive, as the individual created will be free from mitochondrial disease from the moment of conception.

MST was performed in primates (*Macaca mulatta*) in 2009, resulting in the birth of four healthy monkeys, in which the presence of maternal mitochondria was not detected, with a sensitivity of 3% [28]. These were the first animals born following an MST procedure. The technique was then tested in human eggs, and although 52% of the zygotes were abnormally fertilised, the rest were able to develop to blastocysts and produce stem cells in a manner similar to the controls [29]. So far, the efficiency of the technique has improved to reach a carryover less than 1% [26]. However, Yamada et al. and Kang et al. observed that, despite the low levels of mtDNA carryover, there was sometimes gradual loss of the donor mtDNA and re-establishment of the maternal haplotype [24,26].

In April 2016, the first child resulting from this technique was born in Mexico [39]. The mother was an asymptomatic carrier of a mitochondrial mutation that caused Leigh syndrome, a fatal neurological disorder. Despite the fact that she did not have the syndrome, the disease could be transmitted to her children and, in fact, she had suffered four miscarriages and had two children with the disease, who died at the ages of six years and eight months, respectively. The child, who has 1% of its mother’s mtDNA, was healthy at three months, although it is not known if any abnormality might appear in the future.

#### 2.1.3. Polar Body Transfer

A potential new technique for mitochondrial replacement, PBT, was described in a 2014 publication [31].

The first polar body (PB1) is formed during egg maturation. In this process, the DNA duplicates, so that the egg contains four chromosome sets. Of these, two will remain within the egg, while the other two will package, forming the PB1, which is extruded and will not be present in the resulting embryo. The second polar body (PB2) is formed during fertilisation. One set of the remaining chromosomes is packaged, forming the PB2, while the other set will form the nuclear DNA of the embryo together with the sperm DNA.

**Figure 3:**
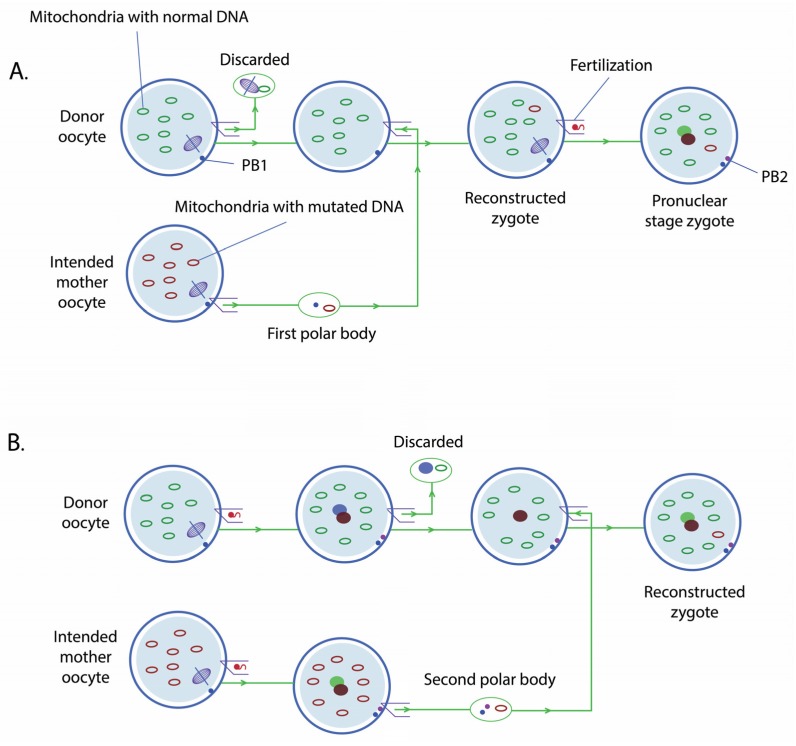
(**A**) first polar body transfer; (**B**) second polar body transfer.

Polar bodies contain very few mitochondria, which is an advantage for avoiding mitochondrial carryover. PBT consists in transferring the PB1 to an unfertilised enucleated donor egg (PB1T) or the PB2 to a half enucleated zygote (PB2T) (Figure 3). Thus, the first strategy is strictly preventive, while the second is not.

Wang et al. compared PB1T with MST and PB2T with PNT [31]. With regard to the developmental outcomes, they found that the rate of embryos developing to the blastocyst stage was the same for PB1T and MST (87.5% and 85.7%, respectively), while PB2T was less efficient than PNT (55.5% and 81.3%, respectively). The number of live births was also similar to those they obtained for unmanipulated embryos (control). Regarding donor mtDNA carryovers in the F1 and F2 offspring, they found that mtDNA carryover with PNT was much higher than with PB1T, MST and PB2T, which achieved low or undetectable mtDNA carryover. This could be due to the mtDNA amplification around pronuclei that occurs in zygotic activation [31], or to the inexperience of Wang et al. with this technique [40].

### 2.2. Mitochondrial Gene Editing

#### 2.2.1. CRISPR/Cas9

CRISPR/Cas9 was identified by Mojica as a natural system that provides bacteria with an adaptive response against viruses [41]. In 2012, Doudna and Charpentier published a study in which they detailed how this system could be used to perform programmed gene editing in different cell types [42]. The method consists in using a custom single guide RNA (sgRNA) fragment that has a dual function. On one hand, it acts as a guide to find the piece of DNA to be modified and binds to it. On the other, it recruits the enzyme Cas9, whose function is to cut the DNA. This enables the desired pieces of DNA to be cut, allowing the modification or removal of specific sequences. Unlike other gene editing methods, CRISPR-Cas9 is cheap, easy to use and very efficient, with the result that its use has become widespread in laboratories throughout the world within a very short period of time.

We are only beginning to glimpse the enormous possibilities offered by this new biotechnology tool, which as well as a multitude of applications in the medical field, has environmental, agricultural and livestock applications [43]. The healthcare applications arouse most interest due to their direct impact on people’s lives, and at the same time the most controversy, mainly in relation to germline genetic modification (gametes and embryos).

Although there is considerable reluctance among the scientific community in the use of this technique in the germ line [44,45], studies using non-viable embryos have already been carried out [46,47]. These studies show that the technique worked with a low efficiency of on-target gene modification and that it generated off-target mutations and mosaicism in the embryos.

CRISPR/Cas9 has already been successfully employed to produce mitochondrial sequence-specific cleavage, as a proof of concept of the potential of this technique to target specific mitochondrial genes [48]. In the same work, researchers engineered a new version of the enzyme Cas9, mitoCas9, whose localization is restricted to mitochondria matrix. This is highly relevant, since it would reduce the risk of off-target mutations in the embryos and prospective children.

#### 2.2.2. TALENs

TALENs are engineered nucleases composed of a transcription activator-like effector DNA-binding domain from Xanthomonas fused to a FokI nuclease domain [49]. When the action of the TALENs is directed specifically at the mtDNA, they are called mito-TALENs [33], which can be used to cleave the mutated mtDNA. In this respect, TALENs has already been used to selectively eliminate defective mtDNA in both unfertilised mouse eggs and in murine embryos; moreover, these genetically modified mice also gave birth to two successive generations of healthy mice [50]. When an mRNA encoding mito-TALENs was injected in an oocyte from a heteroplasmic mouse model carrying two different mtDNA haplotypes (NZB and BALB), mtDNA heteroplasmy shift was achieved. Furthermore, mito-TALENs successfully targeted and reduced the levels of human mtDNA mutation when injected in human patient cells fused to mouse oocytes.

## 3. Bioethical Issues

Techniques involving mitochondrial transfer raise a series of bioethical issues. Firstly, a significant increase in the number of egg donors would be required to conduct the necessary research and for its clinical application. It has been proposed that in order to manage this increase, a regulation would have to be implemented that would guarantee the donor’s well-being, through proper recruitment and support and a limitation on the number of donations per donor that protects them against the negative effects of repeated ovarian hyperstimulation [14]. In this regard, Baylis argues that there is a risk of coercion and exploitation in disadvantaged women [51]. Although we consider this as something to be taken very much into account, it does not constitute an ethical problem intrinsic to mitochondrial replacement techniques, but rather a consequence dependent on the human individuals involved, which must be strictly regulated.

We believe that the ethical issues really intrinsic to the clinical use of these techniques stems mainly from the fact that they involve a genetic modification of the germline, and that children born following their use would have a genetic link to three people: their parents and the donor. We must also consider the fact that it is currently planned to extend the application of these techniques to the field of infertility treatment. Thus, what it was first proposed as an exceptional application in the case of mitochondrial diseases, once it has gained acceptance, the intention would be to extend it to other fields, giving weight to the “slippery slope” argument used by those opposed to these techniques: that once the door has been opened to germline genetic modification, it is merely a matter of time until its use is extended to various applications [52].

### 3.1. Germline Genetic Modification

Genetic modification of the germline occurs when foreign DNA is introduced into the gametes or early embryo, which will pass to any children and, therefore, future generations. While somatic gene modification is generally accepted, since it does not alter the genome overall and is not transmissible to offspring, germline gene modification is more controversial. In this case, the risks of the genetic modification are very difficult to predict, exacerbated by the fact that the modification will be transmitted to the offspring. Furthermore, persons born following the application of these techniques cannot give their informed consent, and germline genetic manipulation could be used for human enhancement instead of therapeutic purposes.

Nevertheless, there is no general consensus among investigators as regards including these techniques in the field of germline gene modification [53]. The main argument against this inclusion is that the nucleus remains intact, and that no genome is modified, but whole mitochondria are replaced [14]. It is the donor mtDNA that, in different proportions according to the technique, will appear in the individual and in subsequent generations. Therefore, in principle, current ethical restrictions for modification of nDNA would not apply. However, the fact is that it is the nuclear genome that is transferred and replaced, so some authors have claimed that the term “mitochondrial replacement” or “mitochondrial transfer” is used to intentionally misguide the public debate [54,55,56]. Intentionally or not, the truth is that these terms do not faithfully reflect what is carried out in these techniques, not only because it is the nDNA that is actually transferred, but also because that nucleus is introduced into an egg or zygote which, as well as other mitochondria, has many other cytoplasmic factors of which very little is currently known, apart from the fact that they are critical for early embryonic development [57].

Another argument is that mtDNA in humans contains only 37 genes, approximately 0.1% of the genome [14]. However, oocytes can contain around 200,000 copies of the mitochondrial genome, which is 50% of the total amount of DNA [58]. In addition, if the number of genes are taken into account, more are modified with these techniques, since in the therapy directed to the nucleus a single gene or a few are modified, surely less than 37. In addition, the Y chromosome contains only 86 genes, but its modification would be considered ethically relevant, which leads to think that what is important is the function of these genes [14], which, in the case of mitochondrial DNA, is not known in depth, as explained later.

Finally, mtDNA does not follow Mendelian inheritance, but is transmitted exclusively via maternal means, the reason why the males would not transmit the modification. For this reason and for the aforementioned, Newson and Wrigley propose the term “conditionally inheritable genomic modification” (CIGM) to classify these techniques [53].

In our view, establishing this new category is not correct insofar as it seeks a conceptual dissociation from germline gene modification, which, in our opinion, is any genetic modification that, being carried out in gametes or embryos, will affect all the cells of the resultant organism, which is fulfilled in the case at hand. The method, inheritance mechanism, and type and degree of change are only associated factors that do not affect this definition. Likewise, the fact that it is transmitted to later generations is only a consequence that until now was fulfilled in all cases. In the same way, the Nuffield Council on Bioethics states that it will “refer to the techniques […] as ‘germline therapies’ because they introduce a change that is incorporated into the (mitochondrial) genes of the resulting people, and so will be incorporated into the germline that they will go on to develop [14].

However, the really important question is whether the modification of mtDNA can be considered an event of sufficient genetic importance. Certainly, there is an objection to the establishment of a strict dichotomy between nDNA and mtDNA. The view that mitochondria are responsible only for the production of cellular energy, so that its modification would not pose the same ethical disadvantages as the modification of the nDNA, which could alter essential characteristics of the individual [59], is challenged by evidence that suggests that mtDNA may have a relevant effect on the phenotype, influencing our personal identity. This is due to the close interconnection between nDNA and mtDNA, which has become highly specific over evolutionary time [60].

Thus, one study reported the involvement of mtDNA in cognitive functioning in mice [61], while another detected associations between the mtDNA variant and susceptibility to alcoholism [62]. Another study showed that mice bred so that their nDNA and mtDNA came from different strains tended to age with better health than mice whose nDNA and mtDNA corresponded ancestrally [63]. This study suggest that the mtDNA variant in the individual has a great effect on its interaction with the nDNA, which, in turn, affects the synthesis, functionality and half-life of the mitochondrial proteins, oxidative stress, insulin signalling, obesity and the parameters of aging, including telomere shortening and mitochondrial dysfunction, resulting in profound differences in longevity. In other studies with both vertebrate and invertebrate models, these novel combinations have led to negative health effects [60]. In mice, altered respiration and growth in hybrid cell lines [64], reduced male exercise ability and growth [65] and reduced learning and explorative behavior in males [61] have been reported. In fruit flies, the negative health outcomes of mitochondrial replacement include altered male nuclear gene expression [66], altered male aging [67,68], altered female aging [69,70], male infertility [66,71], altered male fertility [72], altered male and female juvenile viability [73] and impaired mitochondrial function [74]. In seed beetles, different studies show altered egg-to-adult development time [75], altered metabolic rates [76], altered male fertility [77] and altered survival in females [78]. Lastly, reduced juvenile viability and reduced mitochondrial function and ATP production has been reported in copepods [79]. Therefore, novel combinations of nDNA and mtDNA occurring in MRT may be mismatched, that is, may not be fully compatible, which can lead to health complications. In our opinion, each specific case should be examined, weighing the possible positive and negative consequences of the application and/or omission of treatment. Thus, the severity of the disease, the probability of transmitting it and the risks derived from the technique must be considered. It is therefore essential to have as much information as possible about the symptoms of the disease, mechanism of transmission of the mtDNA to offspring and the interaction between the mtDNA and nDNA to determine—in addition to the safety problems that could arise with mismatch of these two genomes—if the mtDNA replacement would have any effect on our identity and, if so, to what extent. Thus, further research is necessary in order to minimize risk to children that would be born after the application of these techniques.

The report of the IOM points out that before clinical studies are started, preclinical research must be conducted, not only on animals but also on human gametes and embryos, since it “might be necessary to learn about and optimize the physical manipulations of oocytes and embryos required for MRT, establish optimal timing for applying the techniques in gamete provider and intended mother gametes, and provide a better understanding of the appropriate application of reagents to achieve desired effects” [32]. The last report to the Human Fertilisation and Embryology Authority (HFEA) carried out by an independent expert panel to undertake a review of mitochondrial donation techniques indicates some research areas of interest, such as whole genome sequencing approaches to investigating mitochondrial disease genetics, studies in ES cells to investigate mtDNA bottlenecks and the effects of specific variants on mtDNA dynamics in combination with specific nuclear alleles, or to study methods to reduce mitochondrial carryover when performing the techniques [22]. With regard to PBT, which is technically less developed, the panel recommends studies “carried out using normal human oocytes subjected to PBT and the embryos and embryonic stem (ES) cell lines derived from them, to explore whether they develop normally and have minimal carryover of mtDNA […] and an examination of methods to prevent premature activation of oocytes or detect abnormally fertilised oocytes” [40].

In addition, we must examine whether there are other possibilities that are either free from, or have less severe negative effects, in both the medical and moral plane. In this respect, it is important to consider that MST and PB1T act on the egg, while PNT and PB2T require the destruction of one embryo for each embryo produced, which is a considerable and unacceptable ethical difficulty for many. The report of the IOM to the FDA states that “In addition to manipulation, MRT would involve the creation and possible destruction of embryos, both in the research phase and in clinical use. The ethical, social, and policy concerns surrounding the creation and destruction of embryos are long-standing […]. The manipulation, creation, and destruction of embryos are opposed by a range of groups, and federal funding for research involving these processes is severely restricted […]. Religious, ethical, social, and policy issues are associated with the creation, manipulation, and destruction of human embryos.” ([32], pp. 102–106).

Taking this into account and given that the terms of safety and efficacy available data do not indicate whether one technique is preferable to the other [22], we think that, on ethical grounds, there is a strong reason to prefer MST and PB1T over PNT and PB2T. In fact, the couple who had the first child born of these techniques [39] chose MST for this reason, since they are Muslims [80].

### 3.2. Application of Mitochondrial Transfer in Cases of Infertility

The factors contained in the cytoplasm of the oocyte are crucial for embryonic development, such that they may be involved in certain fertility problems [57]. Specifically, it has been found that mitochondrial dysfunction is related with various fertility problems [81]. Hence, almost 20 years ago, a technique was developed to make it possible for women who had poor embryonic development and repeated failures of the embryos implantation to have children: so-called ooplasmic transfer [82].

Although the exact mechanism by which this technique contributes to the correct development of the pregnancy is unknown, it is thought that it acts by “rejuvenating” the eggs of infertile women, as it provides better quality cytoplasmic factors, such as mtDNA, mRNA, proteins and other molecules [83]. In 1997, the first baby resulting from this technique was born [84], and by 2001, around 30 had been born [14]. However, some security issues appeared. Two foetuses conceived after the application of the technique were affected by Turner’s syndrome and were aborted, one spontaneously and the other induced [85], and a born child was diagnosed with pervasive developmental disorder (PDD) at 18 months [86]. 

After the publication of these cases, in 2001, the United States Food and Drug Administration banned the practice of this technique. However, it is offered in other countries, such as India, Turkish Republic of Northern Cyprus, Ukraine, Armenia, Georgia, Israel, Turkey, Thailand, Singapore, Germany and Austria [14].

The advent of the new mitochondrial replacement techniques has led to renewed interest in this approach to infertility, giving rise to a lively debate in the scientific community [87].

In relation to this, the company Ovascience (Waltham, MA, United States) offers its Augment treatment, which is designed to improve a patient’s egg health, when it is compromised due to poor egg quality, age or other reasons. In this treatment, mitochondria from a patient’s own immature egg cells are obtained from an ovarian tissue sample and added to the patient’s mature eggs along with sperm during in vitro fertilisation (IVF). In 2013, the first child resulting from this technique was born [88].

Another possibility is to use donor mitochondria, as in the mitochondrial transfer techniques. Two Ukrainian women with fertility problems but with healthy mitochondria have already become pregnant using this method [89]. Similarly, Zhang et al. used PNT to achieve a pregnancy in a woman who had had two failed IVF cycles, achieving a triplet pregnancy, none of which reached full term [38]. 

One central question to be resolved is whether mitochondrial replacement really improves the health of the egg, thus improving fertility. As regards Augment, no animal studies with a control group have been done to determine the efficacy of this technique in improving fertility, or its safety for offspring [87]. With respect to mitochondrial transfer, Cohen, one of the doctors who participated in the development of ooplasmic transfer points out that this cannot be emphatically affirmed. Since only a small number of women participated in his studies, there was no control group and the ooplasm contains many other factors in addition to the mitochondria, which could explain the successful cases [87]. In fact, in the UK, the use of mitochondrial replacement techniques has been approved in cases of mitochondrial disease only and not to treat infertility.

When the ethical and safety problems of these techniques have not yet been resolved, they continue to take steps forward in its clinical application [90], strengthening the slippery slope argument. The fear is that boundaries for use of these techniques would continue to be eroded. Françoise Baylis states: “It provides scientists with ‘a quiet way station’ in which to refine the micromanipulations techniques essential for other human germline interventions (including nDNA germline modification) and human cloning” ([56], p. 12). Thus, research in this field approaches us to the possible modification of the nDNA in the germinal line not only conceptually, but also technically, which could eventually culminate in the production of “designer babies”. In an earlier paper, the author suggests that these techniques may be used for creating genetic ties in lesbian couples [51].

This is related with a second central question, which is whether the two objectives of reproductive medicine involving germline genome editing, i.e., infertility treatment and disease prevention, are ethically the same [91]. We think that the reasons why the use of these techniques is generally wanted to be limited, for the time being, for disease prevention [23], are not ethical in nature, but have more to do with safety concerns. Limiting its application to these concrete cases implies that if things go wrong, the number of affected individuals will be much lower than if their use had also extended to cases of infertility, which are much more common. However, if the safety of these techniques is proven, there is no doubt that their use will be generalized to other fields, as this is already happening nowadays. Certainly, if their safety and efficacy were proven, it would be difficult to deny treatment to some and to allow it to others on ethical grounds, since in both cases it is not a matter of curing a patient but of producing a new individual.

### 3.3. Donor-Recipient Relationship

In terms of the donor–recipient relationship, as mitochondria have their own DNA, their donations have implications not seen in organ or tissue donation, since any children conceived will have a genetic link with three people: their parents and the donor. Today, only a few children have been born following ooplasmic transfer, a technique which consists of adding ooplasm (with its mitochondria) from a young healthy donor to the eggs of a woman with fertility problems. There is no evidence that these people have attempted to establish any type of relationship with their donors or vice versa. However, given the small number of cases, these data do not have great importance [14].

Thus, only assumptions can be made about the consequences that being genetically related to three people will have for the child. In 2014, BBC News introduced a girl, named Alana Saarinen, who was born from ooplasmic transfer, and stated that she would not consider the donor of her mitochondria a third parent [92]. In fact, taking into account the minimal proportion of DNA contributed by the mitochondria (0.1%), it does not seem reasonable to consider the donor as a third progenitor (or a second mother). Therefore, this term does not seem correct for referring to the donor. Moreover, calling the donor “mother” could be harmful for the child, since it could affect the development of their personal identity and their perception of the parental unit.

As regards the child’s possible interest in contacting the donor or vice versa, this is something that could happen, as occurs in other cases of organ, tissue or gamete donation. Thus, mitochondrial donation techniques must be legally regulated so that matters of confidentiality and possible contact with the donors are guaranteed.

One potential way of avoiding this problem is to apply the new gene editing techniques (CRISPR and TALENs) to the mitochondria, in the unfertilised egg (which would be a preventive approach) or in the already created embryo (which would be a curative approach). This would involve correcting the mitochondrial mutation by eliminating the mutant gene and replacing it with the correct one. In this case, the contribution of a donor would not be necessary, so the child would have only its parent’s DNA. Nevertheless, it still remains germline editing.

## 4. Conclusions

It is important to consider that not all the techniques used or proposed in the prevention of mitochondrial disease have the same ethical implications. Thus, within the mitochondrial replacement techniques, MST and PB1T act on the female gamete, while PNT and PB2T entail the destruction of one embryo for every healthy embryo produced, so from an ethical point of view, we consider that the first two are more advisable. This consideration is also effective in the case of the use of mitochondrial replacement to resolve fertility issues. Apart from these differences, the safety evidence up to now is far from reassuring in all cases.

Moreover, gene editing techniques do not require the intervention of a donor with healthy mitochondria, which avoids the problem of the genetic link of the individual with three persons, and with the legal and ethical problems that this entails.

From our perspective, studies must be conducted in animal models on mitochondria–nucleus communication, transmission of mtDNA to the offspring—especially as regards the variable transmission of heteroplasmy due to the bottleneck effect—and symptoms of mitochondrial diseases, before we can successfully undertake these techniques. Since animal studies have limitations in predicting outcomes in humans, subsequent research in humans would be necessary.

It therefore does not seem prudent to continue moving forward in the application of mitochondrial replacement techniques in humans, nor for infertility treatment nor for disease prevention, when there is still so much to discover about the biology of mtDNA, more so when the intention is not to treat sick people, but to produce new individuals in vitro. We therefore propose a moratorium on their use in humans until we have gained an in depth understanding of the biological mechanisms involved.
